# Magnetic resonance imaging in glioblastoma radiotherapy − beyond treatment adaptation

**DOI:** 10.1016/j.phro.2025.100754

**Published:** 2025-03-20

**Authors:** Viktor R. Paczona, Zoltán Végváry, Gyöngyi Kelemen, Ágnes Dobi, Emőke Borzási, Linda Varga, Adrienne Cserháti, Angéla Csomor, Bence Radics, Sándor Dósa, Márton Balázsfi, Emese Fodor, Ferenc Borzák, Árpád Puskás, Zoltán Varga, Judit Oláh, Katalin Hideghéty

**Affiliations:** a Department of Oncotherapy, University of Szeged. 12 Korányi fasor, Szeged 6720, Hungary; b Department of Radiology, University of Szeged. 6 Semmelweis utca, Szeged 6720, Hungary; c Department of Pathology, University of Szeged. 2 Állomás utca, Szeged 6725, Hungary; d Department of Neurosurgery, University of Szeged. 6 Semmelweis utca, Szeged 6720, Hungary

**Keywords:** Glioblastoma, Adaptive radiotherapy, ART, Magnetic resonance imaging, MRI, Glioma

## Abstract

**Background and Purpose:**

The treatment of glioblastoma remains a challenging task for modern radiation oncology. Adaptive radiotherapy potentially improves local control and reduces toxicity to healthy brain tissue. The purpose of the study was to examine the safety of adaptive radiotherapy in glioblastoma, using a margin-reduction approach based on an interim magnetic resonance image (MRI). Furthermore, it aimed to identify radiomorphological features that may correlate with disease outcome.

**Materials and Methods:**

108 glioblastoma patients receiving standard chemoradiotherapy underwent repeated MRI after 40 Gy. The images were compared to the pre-radiotherapy MRI, based on the following criteria: midline shift, perifocal edema, contrast enhancement, ventricular compression, new lesion outside the radiation field, gross tumor volume (GTV) and planning target volume (PTV) size. Target volumes were adjusted by taking into consideration the new intracranial conditions and the remaining 20 Gy was delivered. Statistical analysis consisted of the comparison of the radiomorphological features to overall and progression free survival.

**Results:**

Increased or unchanged contrast enhancement (HR: 2.11 and 1.18 consecutively) and ventricular compression (HR: 13.58 and 2.53) on the interim MRI resulted in significantly poorer survival. GTV size (initial: 61.4 [3.8–170.9], adapted: 45.3 [0–206.8] cm3) reduction (absolute: −16.2 [-115.3–115.5] cm3, relative: −24.5 [-100–258.9] %) also had demonstrable impact on survival. Changes in PTV, however, did not significantly correlate with survival.

**Conclusions:**

By reducing PTV based on an interim MRI, we achieved substantial sparing of critical normal tissues, without compromising survival. The established evaluation categories can facilitate the systematic review of interim MRI findings.

## 1 Introduction

Adaptive radiotherapy (ART), the method of adapting the radiation plan to the anatomical changes occurring over the course of treatment [1], is gaining ground steadily in personalized radiation oncology. This approach may allow improved local control, reduced toxicities [1] and eventually dose escalation in certain tumor localizations. Its usefulness has been confirmed in the treatment of head and neck, lung and prostate cancer [2]. Interestingly, in the past, less emphasis had been placed on its implication in the radiotherapy of intracranial tumors. Recently, the utility of pre-radiotherapy MRI and monitoring changes in target volume have further been validated [3], [4].

The standard treatment for glioblastoma is post-surgery chemoradiotherapy with Temozolomide, a radiation-enhancing cytostatic agent. However, different approaches are employed for target volume delineation and dose-delivery. The “single-phase” approach recommended by the European Organization for Research and Treatment of Cancer (EORTC) and endorsed by the latest ESTRO-EANO guideline [5] consists of a 30x2 Gy external beam radiotherapy treatment course. According to this approach, the initially defined target volumes remain unaltered (gross tumor volume − GTV, clinical target volume − CTV, i.e. GTV plus a security margin of 5–15 mm, planning target volume − PTV; CTV plus a margin of 0–5 mm) throughout the treatment. This method recommends the selective use of T2/FLAIR abnormalities as some are more specific for tumor infiltration (eg. cortex and grey matter infiltration, mass effect or ventricular v compression) than others (eg. oedema following white matter tracts and respecting the cortex) [5]. In contrast, the two-step treatment proposed by the Radiotherapy and Oncology Group (RTOG) begins with the irradiation of a larger brain area (GTV1, CTV1, PTV1) up to 46 Gy, followed by the delivery of the remaining 14 Gy to smaller volumes (GTV2, CTV2, PTV2) [6]. Lately, numerous US-based institutions as well as the Adult Brain Tumor Consortium (ABTC) also advocate for a limited-field approach. Recent trials have shown that omitting T2 hyperintensity and reducing margin expansions does not result in inferior outcomes or alter patterns of failure. [7], [8]. The target volume delineation and fractionation scheme of the MD Anderson Cancer Center (MDACC) reflects this limited-margin approach. At the initial phase, a dose of 40 Gy is delivered, followed by a sequential boost of 20 Gy with volume reduction [8]. The motivation to reduce treatment volumes stems from the goal of minimizing late effects, such as cognitive decline and radiation-induced lymphopenia [9], [10]. Given the trend of decreasing target volumes over the decades, it is imperative to enhance definition accuracy and adapt radiation delivery to the changes that occur during the treatment.

Our institutional protocol is closely aligned with the RTOG scheme but includes two significant differences. First, though the course of treatment is also split into two phases, the transition occurs at 40 Gy rather than 46 Gy. Second, after the first 40 Gy, a repeated MRI scan is performed to assess the anatomical changes that have occurred during the initial phase of the treatment, allowing for adjustments to the target volumes. The modified target volumes then receive the remaining 20 Gy.

During treatment delivery, ionizing radiation induces an inflammatory response in the endothelium of small blood vessels [11], resulting in temporarily increased vascular permeability and contrast media extravasation [12]. Since blood–brain-barrier (BBB) is not present in the blood vessels of the tumor [13], areas containing cancer cells in low volume and tumorous neovasculature may become discernible following radiation exposure, as these vessels tend to let through more contrast agent in comparison to the healthy brain tissue with functional BBB.

The potential benefit of MRI verification of the intracranial status during chemoradiotherapy is that information can be obtained on the biological behavior of the tumor. By interpreting the interim MRI with caution and necessary experience, useful conclusions can be drawn. If unfavorable tumor response is assumable on the images, or unambiguous progression (i. e. new lesion outside the radiation field) is detected, precious time can be saved by modifying the radiotherapy (RT) (i.e. additional stereotactic irradiation of the new lesion) or directing the patient towards second line systemic treatment or repeated surgery. It needs to be noted that any of the listed salvage modalities are potentially eligible. However, since all are lacking high-level scientific evidence, treatment choice must be made on individual base. Apart from evident radiological signs suggesting disease progression, we hope to identify morphological features that may indicate resistance to chemoradiotherapy and might exert an effect on the overall outcome. Furthermore, in case of obvious tumor shrinkage, relevant healthy brain tissue could be spared with PTV reduction.

In a previous pilot study [14] conducted at our Department, we demonstrated that good tumor response during chemoradiotherapy (CRT) had significant impact on the disease outcome. Importantly, repeated imaging-based replanning with target volume reduction resulted in improved overall survival (p = 0.026). These findings prompted further investigation. Consequently, a larger cohort of patients was enrolled, and Gadolinium-enhanced interval imaging was applied.

In addition to highlight the clinical benefit of repeated MR imaging during CRT, we validated a simple 6-point evaluation system to assess key prognostic factors and facilitate adaptation to target volume changes. This structured algorithm aims to enhance the clinical decision-taking process based on interim MRI findings.

## 2 Materials and methods

108 consecutive patients with histologically verified glioblastoma were selected in our retrospective study. The study population received adjuvant radiation therapy with or without concomitant chemotherapy at the Department of Oncotherapy, University of Szeged between July 2018 and March 2024. All the patients had a preoperative brain MRI and underwent an immediate postoperative MRI (<48 h) following surgery. Informed consent was obtained from all the participants. The study was approved by the National Ethic Committee/TUKEB, ethical approval ID: BM/17723–3/2024.

*Target volume delineation and treatment planning:* All enrolled patients were immobilized in supine position using 3-point individual thermoplastic masks. Planning CT was performed on a GE DiscoveryTM 590 RT scanner with slice thickness of 2.5 mm. Pre-and postoperative MRI scans were co-registered to the planning CT, and in 12 cases amino-acid based PET/CT was also utilized. All MRI scans were acquired on 1.5 T/3 T devices (GE Signa^TM^ Artist 1.5 T, GE Discovery^TM^ MR750w GEM), as per routine, according to institutional glioblastoma protocol (for details, see the supplementary material). Operative bed and eventual macroscopic tumor were defined using gadolinium-enhanced T1-weighted scans, while for the nonenhancing portion T2-and/or FLAIR sequences were taken into consideration. Gross tumor volume included visible macroscopic tumor on pre-and post-surgery MRI as well as the surgical cavity.

To obtain the clinical target volume, GTV was extended by 15 mm in all dimensions, constrained at anatomical barriers (bone, falx, ventricles, visual pathways/optic chiasm, cerebellar tentorium and brainstem). CTV was manually adjusted to overlap with the T2/FLAIR high signal intensity areas supposedly containing viable tumor cells. The planning target volume was a further 3 mm expansion of the CTV, accounting for eventual positioning uncertainties. All patients underwent repeated brain MRI at ⅔ of the chemoradiotherapy.

We evaluated the MRI images across six categories. Adjusted target volumes, specifically GTV1 and PTV1 (with PTV1 being a 10 mm extension of GTV1, necessarily adjusted to anatomical boundaries and pathology), were defined. The 10 mm adaptive GTV-PTV margin was inspired by the two-step guideline of the American Brain Tumor Consortium (ABTC) [15]. This workgroup recommends the utilization of a 5 mm GTV-CTV extension for tumor boost, and a further 3–5 mm set-up margin to obtain the planning target volume. However, for the sake of simplicity and easier comparison, adapted CTV and PTV were merged into one volume called PTV1 (i.e. 5 mm for CTV1 + 5 mm for PTV1). When performing the target volume adaptation, all the MRI sequences that were primarily used were re-evaluated and taken into consideration. Based on this interim study, the new contours were created on the initial planning CT, following CT-repeated MRI image fusion.

Prior to each treatment session, cone-beam CT image verification was performed. IMRT planning (VMAT) was exclusively used throughout the study.

After chemoradiation patients received systemic therapy according to the Stupp regimen [16]. The follow up consisted of a monthly onco-neurological examination and brain MRI every 3 months.

*Statistical analysis:* The primary endpoint of our study was overall survival (OS), defined as the period between the histopathological confirmation of the malignancy and the date of the patient’s death. In addition, progression free survival (PFS) was also investigated in certain cases, i.e. the time between the histopathological confirmation of the tumor and the detection of recurrence.

Kaplan-Meier method was utilized in the analysis of categorical variables. If statistical significance was found, Cox proportional hazard regression models were also employed for the given variables, as well as for all the continuous values.

Statistical analysis was conducted using the SPSS statistical analysis software package (version 20; IBM, Armonk, NY, USA). Statistical significance was set at a threshold of p < 0.05. The software used for the graphical representation of our findings was SRplot (https://www.bioinformatics.com.cn/srplot).

## 3 Results

Patient characteristics are summarized in Table 1 and Table 2.

**Table 1:** The numbers indicate the number of patients, if not specified otherwise.

**Characteristic**	
Gender	
- Male	66
- Female	42
Age (years)	55.6 [23–78]^a^
Histology	
- Glioblastoma (IDH1 Wild-Type)	89
- Astrocytoma Grade 4 (IDH1 Mutant) Type of surgery	19
- Biopsy	9
- Partial resection	57
- Gross tumor resection	42
Time interval between surgeryand start of CRT (days)	29.5 [7–61]
Karnofsky performance status prior to CRT	
- > 60 %	101
- ≤ 60 %	7
MGMT promoter methylation status (available in 83 cases)	52
Therapy	
- Chemoradiotherapy	108
First line of treament	
- Temozolomide monotherapy	
Number of recipients	87
Average number of cycles	11.7 [1–72]

a Average, minimum and maximum in square brackets.

**Table 2:** The numbers indicate the number of patients, if not specified otherwise.

**Characteristic**	
Second line of treatment	
- Bevacizumab therapy	
Number of recipients	41
Average number of cycles	16.9 [2–66]
Third line of treatment	
- Lomustine therapy	
Number of recipients	9
Average number of cycles	8.2 [1], [2], [3], [4], [5], [6], [7], [8], [9], [10], [11], [12], [13], [14], [15], [16]
Reoperation	
- Repeated surgery following chemoradiotherapy	3
- Repeated surgery following progression on Temozolomide	19
- Repeated surgery following progression on Bevacizumab	6
Reirradiation	
- Repeated irradiation following progression on Temozolomide	12
- Repeated irradiation following progression on Bevacizumab	8

^a^ Average, minimum and maximum in square brackets.

This study population represents real-world data, with no patient selection throughout the inclusion period. The overall survival (OS) for the entire cohort was 20.7 months, and the disease-free survival (DFS) was 10.7 months. In case of IDH1 Wild-Type tumors, the OS was 18.9 months and the PFS 9.2 months. Patients with IDH1 mutation had better prognosis, i.e. the OS was found to be 28.9 months and the PFS 18.1 months.

Median follow-up time was defined as the period between the last day of CRT and the date of the patient’s death, or his/her last visit. According to this train of thought, the median follow-up time was 19.7 months.

The repeated MRI scans were compared to postoperative, pre-radiotherapy images to assess the changes that occurred within the cranial cavity during the first phase of chemoradiation. For this purpose, a six-point evaluation system was established, which can be seen in Table 3 and Table 4.

**Table 3:** The numbers indicate the number of patients.

**Radiological feature**	**Not present**^a^	**Present, no change**^b^	**Increase**^b^	**Decrease**^b^
Midline shift	47	20	3	38
Perifocal edema	15	20	14	59
Contrast enhancement	2	25	40	41
Ventricular compression	62	22	2	22

a On the pre-RT MRI.

b On the interim MRI.

**Table 4:** The numbers indicate the number of patients.

**Radiological feature**	
Initial ventricular status	
Normal	51
Enlarged	30
Compressed	27
New lesion outside PTV	
Yes	7
No	101

Volumetric changes of the gross tumor volume and planning target volume are summarized in Table 4.

Other changes that were commonly observed on the repeated MRI were miscellaneous findings, such as the resorption of surgery-related blood clot and gelatine foam. However, in one case cerebral abscess was detected and the patient was immediately transferred to the Department of Neurosurgery for surgical intervention. In two other patients with deteriorating neurological status the repeated MRI demonstrated the signs of meningitis and antibiosis was started accordingly (see Table 5.).

**Table 5:** Volumetric changes of the gross tumor volume and planning target volume.

**Target volume**	**Initial volume (cm^3^)**	**Adapted volume (cm^3^)**	**Absolute volume change (cm^3^)**	**Relative volume change (%)**
GTV	61.4 [3.8–170.9]^a^	45.3 [0–206.8]	−16.2 [-115.3–115.5]^b^	−24.5 [-100–258.9]
PTV	347.1 [109.1–811.3]	260 [39.2–582]	−79.2 [-490.5–182.9]	–22.9 [-72.9–90.6]

a Average, minimum and maximum in square brackets.

b Minus (−) sign signifies volume shrinkage.

The presence of contrast enhancement, midline shift and perifocal edema on the pre-RT MRI scans had no effect on the overall survival at all. Change in the two latter did not influence the outcome of the disease either. The initial ventricular status, however, demonstrated significant correlation with the OS, as enlarged (HR: 1.56) and compressed (HR: 3.07) ventricles were associated with unfavorable prognosis. A similar tendency was observed if change in ventricular compression was compared to overall survival. No change throughout the radiotherapy corresponded to a hazard ratio of 2.53. An even more dramatic result was obtained if ventricular compression increased during the treatment, as this phenomenon was associated with a HR of 13.58. The control group was defined as the patient population with no ventricular compression on the pre-radiotherapy MRI.

Though predictable, the decrease in tumor enhancement also proved to correlate significantly with improved life expectancy. Increased enhancement on the repeated MRI was found to pair with a HR of 2.11, while no change in contrast uptake corresponded to a hazard ratio of 1.18, compared to the control group. Similar tendency was observable when contrast enhancement was plotted against progression free survival (PFS). In this case, no significant difference was shown between the control (decreased contrast enhancement on the repeated MRI) and stationary groups, but contrast agent uptake increase was associated with higher hazard (HR: 1.73).

Both absolute and relative tumor volume changes had a significant influence on disease outcome. Absolute volume shrinkage, measured in cubic centimeters, showed a slightly stronger impact on survival compared to relative tumor size reduction, expressed as a percentage (HR: 1.009 vs. 1.007). However, when assessing (PFS), absolute tumor volume change did not reach statistical significance (p = 0.257). In contrast, relative volume reduction demonstrated significant correlation with PFS (p = 0.049), underscoring its potential prognostic value.

In multivariate analysis, tumor volume change, change in contrast enhancement and ventricular status were the four factors that played an independent role in the overall survival.

## 4 Discussion

The use of adaptive radiotherapy in the treatment of glioblastoma is sparsely documented and articles usually report on a small patient population. To our best knowledge, our present study has enrolled the highest number of patients in the topic.

Though the latest guidelines [5] recommend a fresh MRI within 2 weeks prior to the RT start date, it has traditionally been acceptable to use the post-surgery MR exam for RT planning purposes [17], [18]. The average waiting time from the 48 h post-surgery MRI to the planning CT was 20 days, as we were committed to start the adjuvant radiotherapy as soon as clinically possible. This means that we should have performed an extra MRI just one week after the surgery. However, in case of subtotal tumor resection, surgery-related complications on the postoperative MRI or extended convalescence, a new MRI was always performed before the RT planning.

In a recent research [19], MRI-Linac was utilized for weekly re-planning of glioblastoma patients. The daily T2–weighted images proved to be sufficient to track the shrinkage of the surgical cavity that occurred in 50 % of the cases. On this basis, it has been concluded that the adaptive approach may prevent radiation-induced neurocognitive deterioration by allowing improved hippocampal and brain sparing. The same finding was made by a Japanese workgroup [20] using single-step replanning. In their study contrast-enhanced T1-weighted MR images were taken at an irradiation dose of 34–38 Gy and the final 20 Gy boost was delivered after the adaptation of the target volume. A Turkish workgroup [21] followed the RTOG scheme in their adaptive trial and performed a repeated MRI after the first 46 Gy. They have pointed out that adaptive boost treatment planning may be beneficial for two primary reasons. Firstly, target volume expansion and consequent underdosage is more likely to occur if the patient only underwent subtotal tumor resection or biopsy. Secondly, adaptive boost planning is also worth considering for patients with gross tumor resection, since this group is inclined to have target volume decrease, mainly due to surgical cavity shrinkage. With the adaptive approach, we may potentially offer more optimized dose coverage.

In other tumor localizations, the most important benefit of adaptive radiotherapy is allowing the delivery of higher doses to the tumor to achieve improved local disease control. Since glioblastoma recurs in about 80 % of cases in the close vicinity of the original tumor site [22], dose escalation beyond 60 Gy would be desirable. However, these attempts are restricted by the dose constraints of the central nervous system. When it comes to the remaining 20 %, it is possible that peritumoral tractography might be a helpful tool in the prediction of the potential recurrence site, based on probabilistic models [23].

Pre-and post-treatment FLAIR volumes corresponding to micrometastasis-containing edema have been identified as a radiologically verifiable prognostic factor for glioblastoma by a study conducted by Matthew D. Garrett and al. [24].

A new strategy that may play a pivotal role in the identification of individual tumoral patterns as well as prognostic and/or predictive factors for glioblastoma patients is the use of MRI-derived radiomics. This method can extract a broad range of microscale textural patterns from MR images, mostly invisible for the human eye. The scanning of the resection cavity and its surroundings [25] is expected to uncover valuable information on the efficacy of the applied antitumoral therapy and to help differentiate treatment-related changes from tumor recurrence.

A recent comparison demonstrated that postoperative chemoradiation according to RTOG/NRG principles did not differ significantly in terms of treatment-related toxicities and efficacy from treatment according to EORTC recommendations. It needs to be mentioned that this study included grade 3 gliomas beside glioblastoma and grade 4 tumors [26].

Our study demonstrates that valuable imaging information can be collected during RT. Based on this data, it can be concluded that both relative and absolute GTV shrinkage serve as prognostic factors for the disease, as they demonstrate positive correlation with survival.

Neither absolute, nor relative PTV changes significantly correlated with survival in proportional hazards regression model, reinforcing the safety of our approach. More crucially, by reducing PTV based on the interim MRI, we achieved substantial sparing of critical normal tissues, particularly the brain, without compromising survival outcomes. This demonstrates that our adaptive margin reduction strategy is not only noninferior to conventional methods, but also more advantageous. With survival rates matching or exceeding those reported in the literature [27], [28], our approach not only preserves survival but also minimizes dose to surrounding healthy brain tissue, making it a highly effective treatment strategy for glioblastoma.

However, it should be noted that the study has limitations due to its single-institution, retrospective nature. The sample size was not prospectively determined and may be insufficient to reveal small differences. Additionally, the lack of control group limits the possibility of drawing definitive conclusions on the accuracy and efficacy of the proposed delineation strategies. The benefit of any intervention, including the ART performed in this study, needs to be tested in prospective trials.

Nevertheless, we have established important evaluation categories that facilitate the systematic review of interim MRI findings. Furthermore, our data supports the use of a limited PTV approach, particularly the PTV1 strategy.

The following morphological features on the interim MRI were identified as risk factors for unfavorable disease outcome: compressed ventricles, increase in contrast enhancement, and smaller absolute as well as relative tumor volume shrinkage. These phenomena shall be regarded as warning signs necessitating stricter follow-up and possibly more frequent imaging. This increased vigilance is expected to shorten reaction time when tumor progression becomes evident, and patients may benefit earlier from potentially effective second line therapy. Monitoring the resection cavity and residual tumor during chemoradiotherapy and adapting the PTV to these changes, enhances dose coverage of the tumor while sparing normal brain tissue. This may contribute to improved therapeutic ratio and eventually better clinical outcome.

## Declaration of competing interest

The authors declare that they have no known competing financial interests or personal relationships that could have appeared to influence the work reported in this paper.
