# Supplementary material for: Magnetic resonance imaging in glioblastoma radiotherapy − beyond treatment adaptation
Source: Phys Imaging Radiat Oncol. 2025 Mar 20;34:100754. doi: 10.1016/j.phro.2025.100754 (PMC11994382; doi:10.1016/j.phro.2025.100754)

Supplementary Table S1: The University of Szeged MRI protocols for brain tumor assessment

(a) On our GE Discovery^{TM} MR750w GEM scanner

Plane/timing	Sequence
Axial	- T2-weighted fast spin echo (Ax T2 FSE)<br>- T1-weighted fast spoiled gradient echo (Ax T1 3D FSPGR)<br>- diffusion-weighted (Ax DWI)<br>- diffusion tensor based on 25 directions (Ax DTI)<br>- 3D T1-weighted inversion recovery (IR)-prepped fast spoiled gradient echo: 3D Ax T1 FSPGR BRAVO
Sagittal	- IR-prepped T2-weighted 3D fast spin echo (T2 FLAIR CUBE)
Post-contrast	- Axial T1-weighted fast spoiled gradient echo (Ax T1 3D FSPGR)

(b) On our GE Signa^{TM} Artist 1.5T scanner

Plane/timing	Sequence
Axial	- T2-weighted PROPELLER (Ax T2 PROPELLER)<br>- T1-weighted fast spoiled gradient echo (Ax T1 3D FSPGR)<br>- diffusion-weighted (Ax DWI)<br>- diffusion tensor based on 25 directions (Ax DTI)<br>- 3D T1-weighted inversion recovery (IR)-prepped fast spoiled gradient echo: Ax T1 FSPGR BRAVO
Sagittal	- IR-prepped T2-weighted 3D fast spin echo (T2 FLAIR CUBE)
Post-contrast	- Axial T1-weighted fast spoiled gradient echo (Ax T1 3D FSPGR)

Supplementary Figure S2: Patient with complete tumor response on the interim MRI

July 2022: surgical resection. Histology: Glioblastoma, IDH wild-type

Post-surgery MRI:

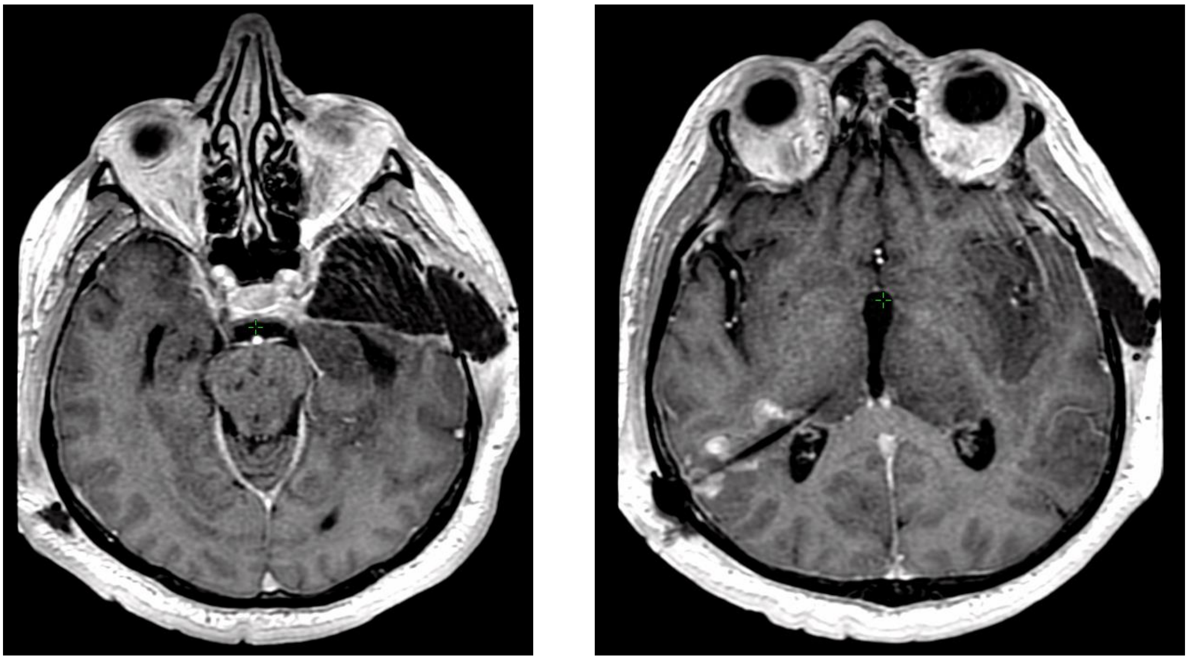

Chemoradiation: August 2022-October 2022

Repeated MRI after 20x2 Gy:

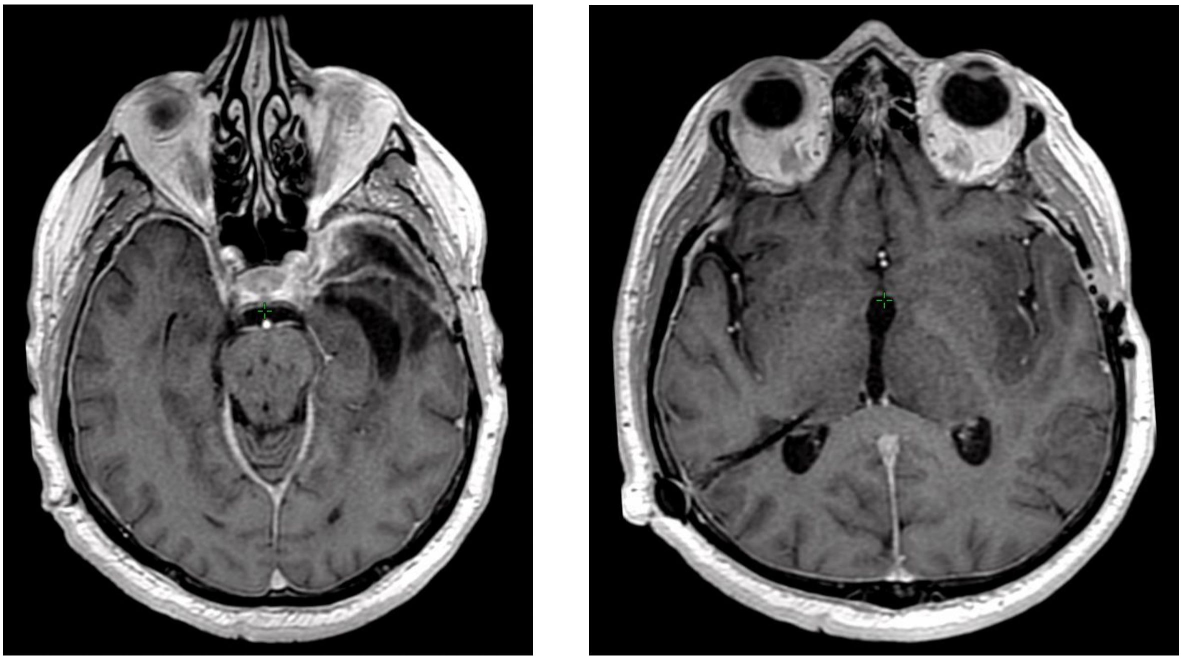

November 2022-July 2023: 9 cycles of temozolomide monotherapy

July 2023: staging MRI reveals progressive disease in the left thalamic region:

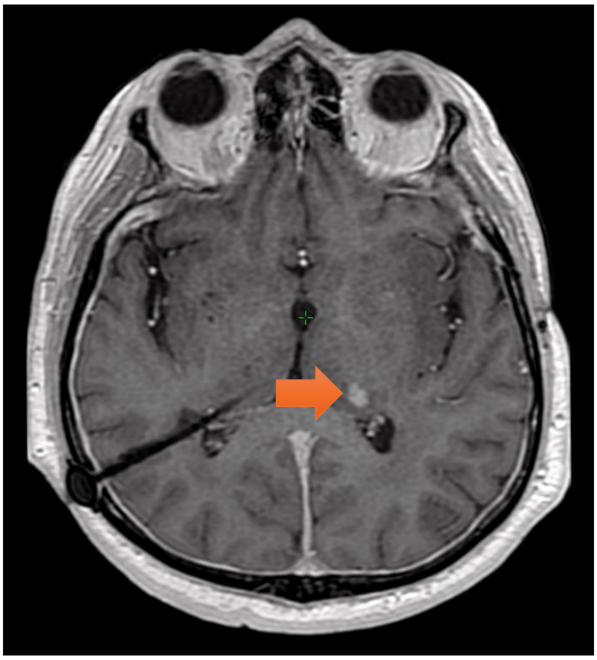

July 2023-August 2023: stereotactic irradiation of the new lesion. Dose: 3x7 Gy

September 2023-: VEGF-I monotherapy (26 cycles until December 2024)

December 2024: no sign of recurrent disease on MRI or 18-F DOPA PET/CT

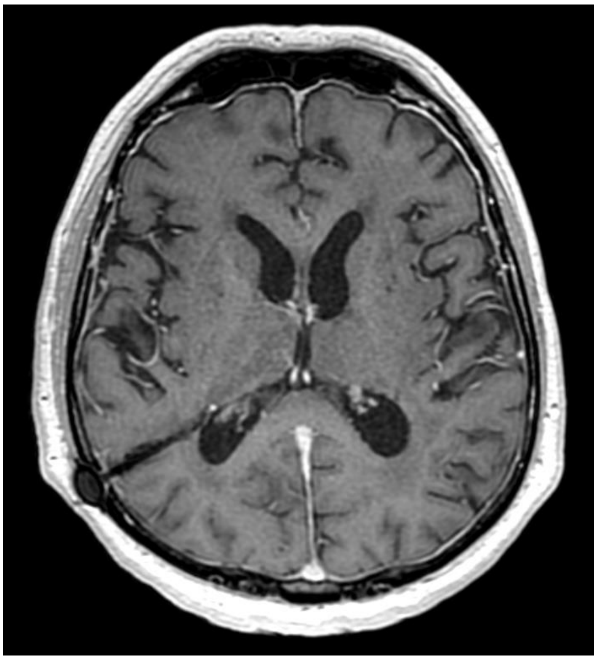

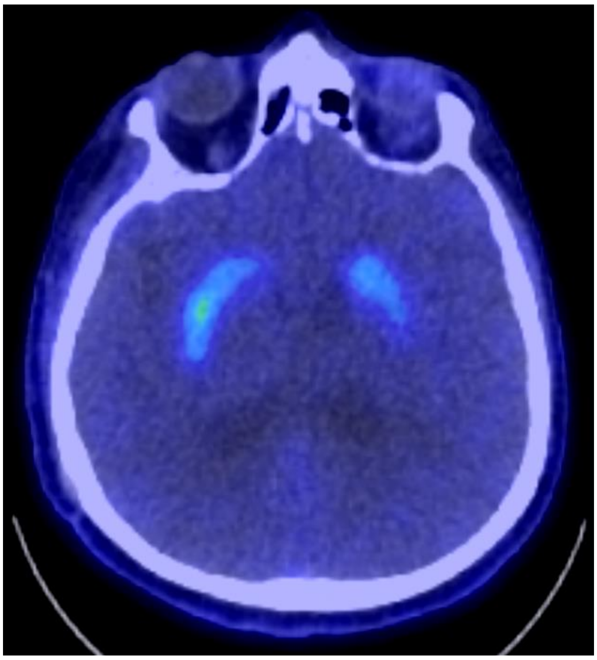

Supplementary Figure S3: Graphical illustration of the adaptation procedure
(a) Initial target volumes on the planning MRI: yellow contour: initial GTV, red contour: initial PTV.
(b) Modified target volumes based on the interim MRI: cyan contour: adapted GTV (GTV1), magenta contour: adapted PTV (PTV1).

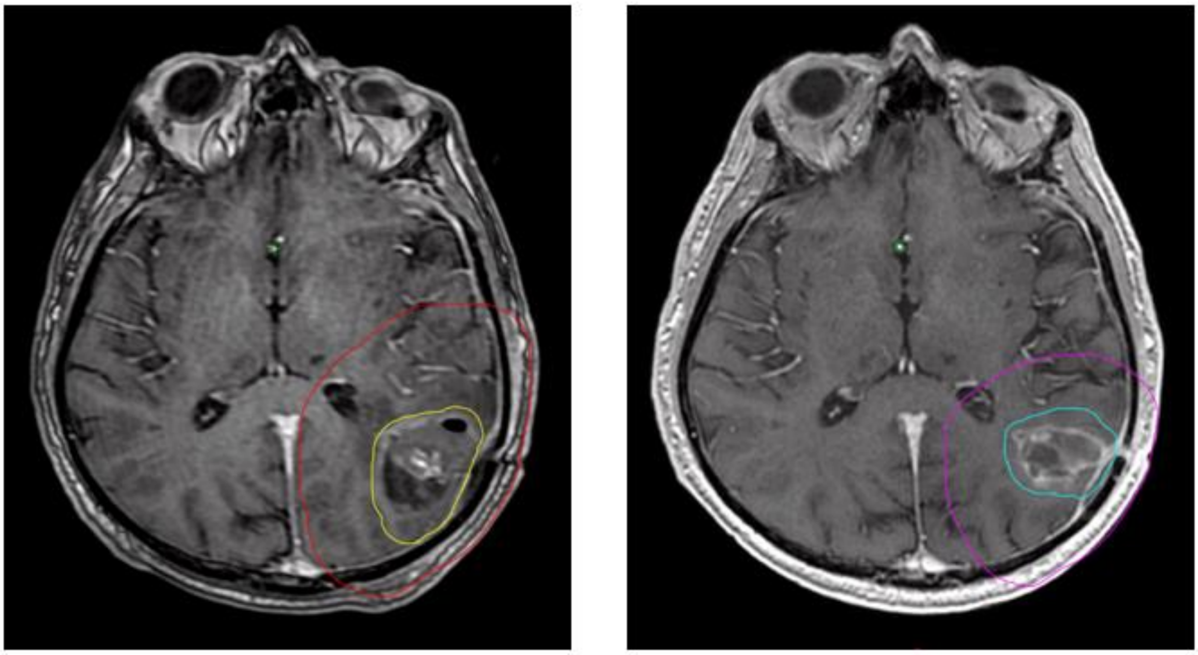

(a) (b)

Supplementary Figure S4: Graphical illustration of the statistical analysis

(a) Initial ventricular status plotted against overall survival.

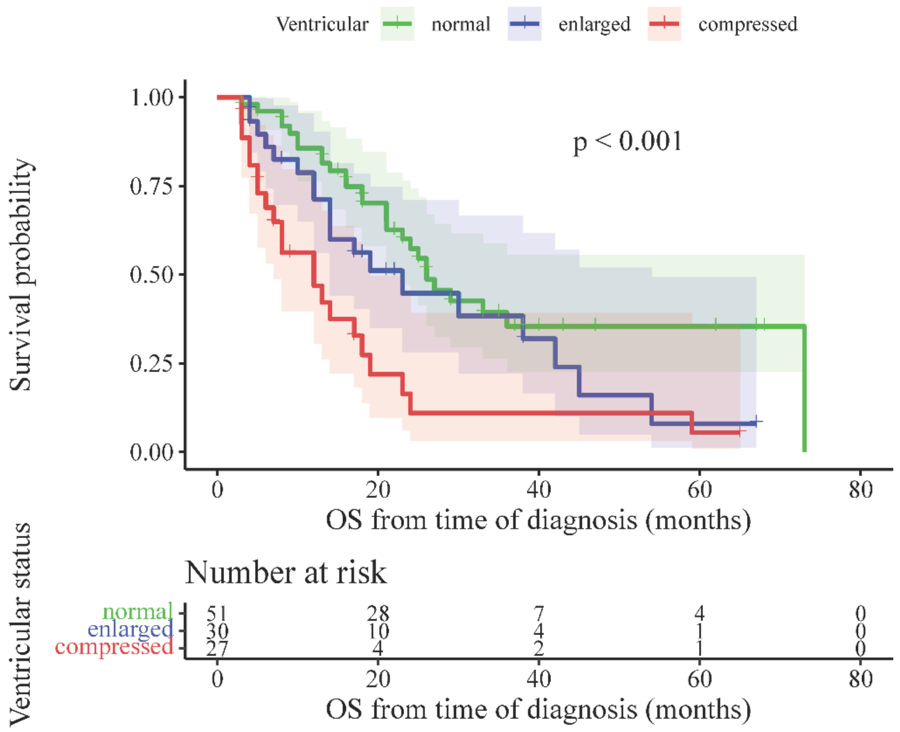

(b) Contrast enhancement change vs. overall survival.

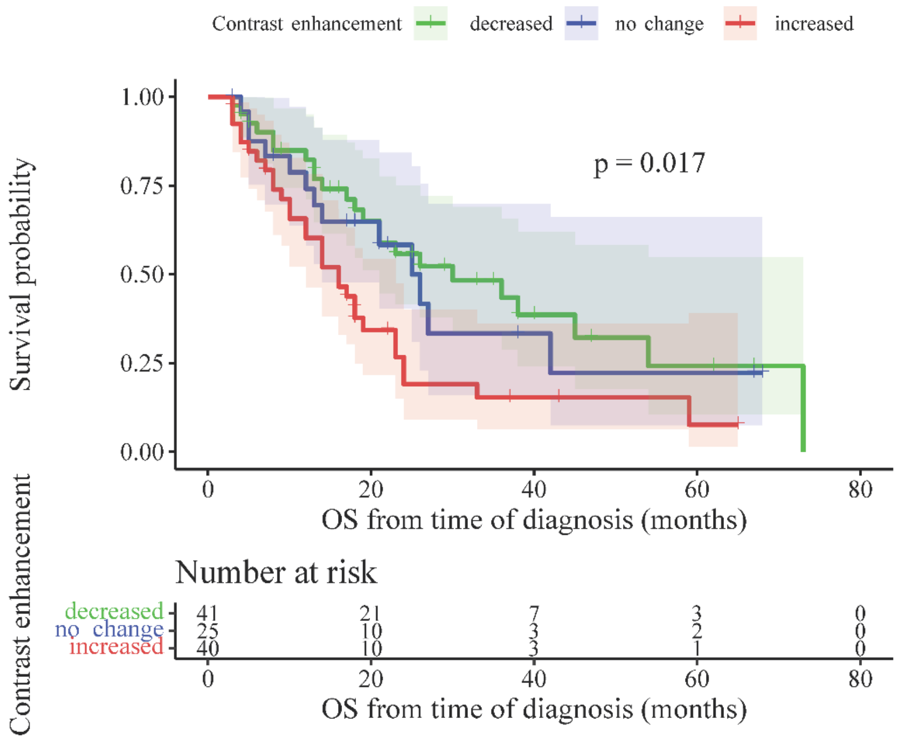

(c) Relative gross tumor volume change vs. overall survival. Three categories were created based on the percentage of volume shrinkage, as indicated on the graph.

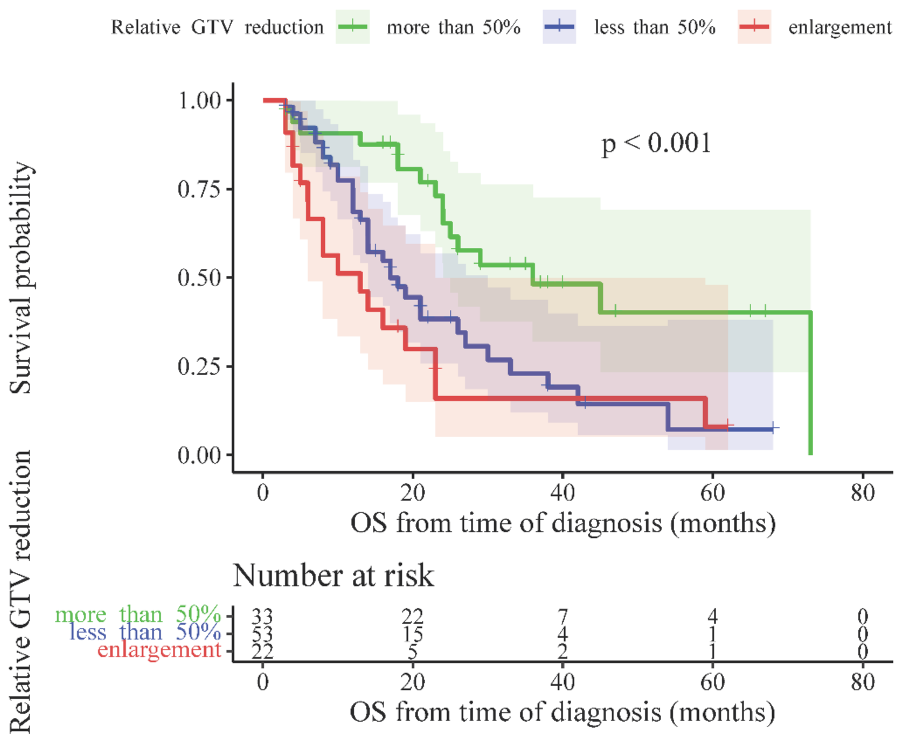

Supplement: Supplementary Data 1 [file mmc1.pdf]
